# Reconstructing Generalized Logical Networks of Transcriptional Regulation in Mouse Brain from Temporal Gene Expression Data

**DOI:** 10.1155/2009/545176

**Published:** 2009-01-27

**Authors:** Mingzhou(Joe) Song, Chris K Lewis, Eric R Lance, Elissa J Chesler, Roumyana Kirova Yordanova, Michael A Langston, Kerrie H Lodowski, Susan E Bergeson

**Affiliations:** 1 Department of Computer Science, New Mexico State University, Las Cruces, NM 88003, USA; 2 Systems Genetics Grooup, Biosciences Division, Oak Ridge National Laboratory, Oak Ridge, TN 37831, USA; 3 Department of Applied Genomics, Bristol-Myers Squibb R&D, P.O. Box 5400, Princeton, NJ 08543, USA; 4 Department of Computer Science, University of Tennessee, Knoxville, TN 37996, USA; 5 Department of Pharmacology, School of Medicine, Case Western Reserve University, Cleveland, OH 44106, USA; 6 Department of Pharmacology and Neuroscience, Texas Tech University, Lubbock, TX 79430, USA

## Abstract

Gene expression time course data can be used not only to detect differentially expressed genes but also to find temporal associations among genes. The problem of reconstructing generalized logical networks to account for temporal dependencies among genes and environmental stimuli from transcriptomic data is addressed. A network reconstruction algorithm was developed that uses statistical significance as a criterion for network selection to avoid false-positive interactions arising from pure chance. The multinomial hypothesis testing-based network reconstruction allows for explicit specification of the false-positive rate, unique from all extant network inference algorithms. The method is superior to dynamic Bayesian network modeling in a simulation study. Temporal gene expression data from the brains of alcohol-treated mice in an analysis of the molecular response to alcohol are used for modeling. Genes from major neuronal pathways are identified as putative components of the alcohol response mechanism. Nine of these genes have associations with alcohol reported in literature. Several other potentially relevant genes, compatible with independent results from literature mining, may play a role in the response to alcohol. Additional, previously unknown gene interactions were discovered that, subject to biological verification, may offer new clues in the search for the elusive molecular mechanisms of alcoholism.

## 1. Introduction

The regulation of transcription occurring in an intriguingly complex biological system involves multiple interacting regulatory processes in gene regulatory networks (GRNs). Modeling transcriptional regulation requires algorithms that retain information about regulatory interactions. The generalized logical network (GLN) is a generative model that can be reconstructed from temporal trajectories, for example, from data collected in time-series studies of gene expression. Because these data capture information on temporal antecedence, the approach can be used to develop stronger hypotheses about casual relations among transcriptional events than one would be able to derive from mere correlation analyses. We designed a GLN reconstruction algorithm that differs from previous approaches because it makes use of hypothesis testing on the multinomial distribution to establish directed connections among genes. Our statistical approach allows explicit control of false positives by specifying a desirable alpha level, while other criteria used in network reconstruction, such as the Bayesian information criterion (BIC) used in dynamic Bayesian networks (DBNs) reconstruction and the coefficient of determination (COD) used in Boolean networks (BNs) reconstruction, do not explicitly enforce false-positive rate control.

GLNs also allow more aspects of systems to be studied than other network models by enabling (1) adaptive description for interactions among variables, (2) nonlinear interaction patterns, and (3) finite steady states, attractor basins, and state transition diagrams. The software CellNetAnalyzer [1] allows a user to draft a GLN from existing knowledge. Our method allows such networks to be reconstructed and derived solely from data-driven approaches. GLNs have the further advantage that they do not require parametric assumptions, unlike stochastic logical networks [2] which discretize differential equations based on strong assumptions. Additionally, our implementation of GLN modeling focuses on network reconstruction from temporal gene expression data, which can be used complementarily with network property analysis algorithms such as the network walking algorithm [3], and literature mining tools such as those reviewed in [4].

GLN is a dynamical system model to characterize interactions among discrete variables over discrete time. It is a directed graph, with nodes representing the discrete variables and each having a generalized truth table (gtt). The gtt for a node  maps all possible combinations of parent node values to values of . Related modeling paradigms with different emphases have also been applied to biological data and are compared and contrasted with the GLN below.

(i) Temporal probabilistic networks. The dynamic Bayesian network (DBN) is an extension of Bayesian networks, which incorporates time transitions between Bayesian networks. A DBN describes temporal statistical dependencies among genes. DBNs have been successful in extracting probabilistic dependencies among genes in GRNs [5–7]. Certain DBNs can even be converted to probabilistic Boolean networks [8]. However, DBN is an indirect tool to understand system dynamics since it does not explicitly describe temporal relations among entities in a functional form, while a GLN provides immediate functional relationships among variables.

(ii) Continuous dynamical system models. Differential equations in both deterministic [9, 10] and stochastic [11] formulations have been used to model interactions in GRNs in continuous time. The E-Cell Project [12, 13] uses differential equations to target knowledge-based reproduction, not data-driven reconstruction, of intracellular biochemical and molecular interactions within a single cell. The stochastic master equations relate state probabilities by differential equations, impractical for biological systems involving many variables because of the computational burden. Recent research has been focusing on improving the scalability of such models [14].

(iii) Discrete dynamical system models. The Boolean network (BN) [1, 15–18] and its Markovian [19] or probabilistic [20] extensions, where each variable takes the value of either 0 or 1, are 1st-order special cases of the GLN. The dichotomous nature of a BN seriously limits its capacity to discriminate quantitative differences among continuous random variables. As most biological networks are rarely binary, much information is lost. This can be crucial when such differences are more interesting than the mere information of presence (1) or absence (0). In addition, the coefficient of determination criterion used in BN reconstruction does not address the issue of model complexity and goodness of fit.

To summarize, these temporal probabilistic networks do not explicitly describe system dynamics. Continuous dynamical system models, computationally and data intensive and thus often not data driven, are also inconvenient for visualizing state transitions. BNs cannot capture subtle and nonlinear interactions. Details of these and various other major network reconstruction and modeling algorithms can be found in recent reviews [21, 22].

Temporal dependency may reflect causal interactions among processes in a dynamical system, but not always. System modeling may be further complicated by incomplete observations—a situation that is typical for biological experiments. For example, protein concentrations, post-translational protein modification states, and small molecular messengers are missing in a GRN developed entirely from transcriptome data. However, a consistent temporal dependency must arise from a causal interaction, even with incomplete observations. Therefore, statistically significant temporal dependencies among genes and environmental stimuli may still constitute a basis to establish causalities.

We reconstruct GLNs from trajectories of discrete random variables, the abundance of mRNAs, in order to uncover temporal dependencies among genes and environmental stimuli. Temporal dependencies among key genes in response to alcohol in mice are assessed through GLN modeling. The effects of alcohol on functions of gene products and the corresponding effect on gene expression are an active research area, particularly in the inflammatory and neural plasticity processes that result in lasting brain changes in response to alcohol. We believe that the GLN approach will provide highly relevant clues to discover biologically important gene interactions involved in the molecular mechanisms of brain changes in alcoholism. The resulting network model demonstrates the tremendous potential for GLN modeling to provide insight into the diverse molecular mechanisms underlying clinical phenomena such as alcoholism.

The paper is organized into eight sections. The GLN is defined in Section 2. A procedure is given in Section 3 to determine the statistical power of reconstructing a GLN given an experimental design. An algorithm for reconstruction of GLNs based on multinomial testing is described in Section 4. Comparisons of reconstruction accuracy between GLN and DBN modeling are made in Section 5. A microarray experiment for the influence of alcohol on mouse brain gene expression is recounted in Section 6. The GLN modeling result of the GRN in the mouse brain in response to alcohol is discussed in Section 7. Finally, conclusions and future work are given in Section 8.

## 2. The Generalized Logical Network

As a discrete-time and discrete-value dynamical system model, a GLN of  nodes is a directed graph with a gtt attached to each node. Each abstract node can represent information about a molecule, a cell, a species, or a stimulus. The gtt allows a discrete variable to take more than two possible values and to reflect subtle but crucial changes, and encodes precisely the biological mechanisms that the nodes use to interact with each other.

Let node  have  quantization levels ranging from 0 to , controlled by  parents  of  quantization levels, respectively. The gtt  of node  is a function that maps all possible combinations of parent node values to values of . Thus, , the value of  at discrete time , can be computed by (1)

With  parents, the size of  is , exponential in  and posing a memory problem. The generalized logical decision diagram is a space efficient data structure to store a gtt by removing fictitious variables and redundancies, extending the binary decision diagram [23].

The following is an example showing the gtt  of  of 3 levels with two parents of 2 and 3 levels, respectively.

Table 1 represents a complex behavior for  as controlled by  and . The influence of  on  is almost opposite depending on the value of . If , the influence is nonlinear and convex; otherwise, the influence is nonlinear and concave. The size of  is .

**Table 1:** 

		
0	0	2
0	1	0
0	2	2
1	0	0
1	1	1
1	2	0

**Figure 1:**
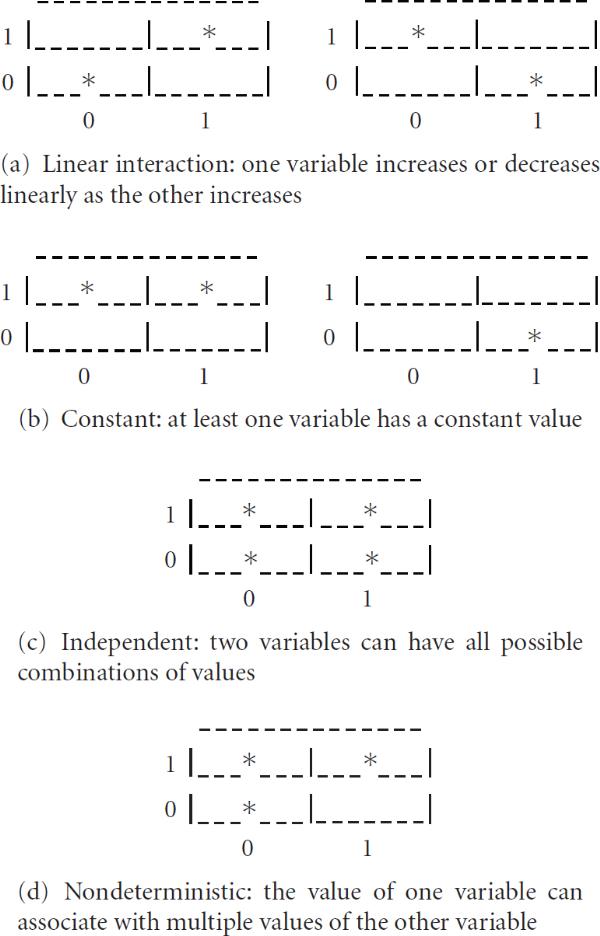
**All possible types of pairwise interaction patterns in a Boolean network**. The rows can be considered the values of one discrete variable and the columns values of another discrete variable. An asterisk (*) represents a co-occurrence of the values in the corresponding row and column. The asterisks together can be considered the interaction behavior of the two discrete variables. Blank cells represent absent values corresponding to the hypothetical interaction pattern.

**Figure 2:**
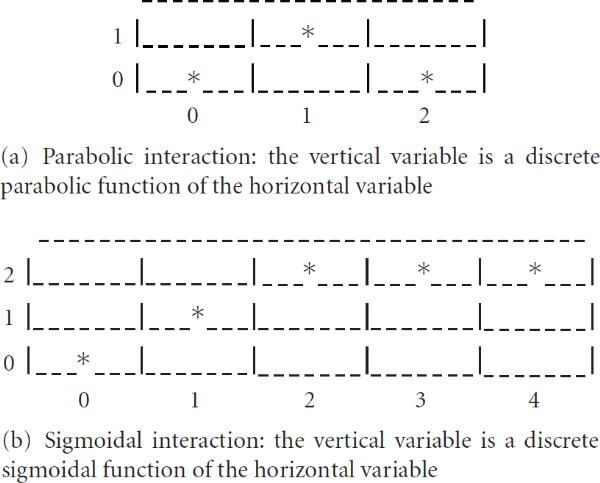
**Two examples of many nonlinear interaction patterns which can be modeled in a generalized logical network, but which are impossible to represent in a Boolean network**. Asterisks represent observed values in the interaction pattern. Blank cells represent absent values corresponding to the hypothetical interaction pattern. The limitation of the Boolean network is due to its incapability of representing the intermediate values, that is, 1 of the vertical variable and 1, 2, and 3 of the horizontal variable in (b).

Such a defined gtt facilitates rich nonlinear interaction patterns. For a comparison, all possible types of pairwise interactions in a truth table of a BN are illustrated in Figure 1; two nonlinear pairwise interactions in a gtt of a GLN are shown in Figure 2, impossible with a BN. It is also worthwhile to point out that a linear correlation-based approach will only be able to detect the linear interactions shown in Figure 1(a), missing all other nonlinear ones shown in Figures 1 and 2.

Let  be the state vector at discrete time (2)

representing the values of all nodes at discrete time . Let  collect the gtts  for all nodes. Let  be the number of parents for each node. The network complexity  of a GLN is the maximum number of incoming edges a node can have, that is,(3)

A GLN is th order if the value of some node at discrete time  involves the parent values from discrete time  through  at most. A synchronous GLN updates the values of all nodes simultaneously through(4)

Synchronous th order GLNs allow modeling of variable time delays abundant in biological systems. Let  be the initial  states of a GLN. A trajectory of length  is defined as . Our discussion is restricted to synchronous and first-order GLNs.

## 3. Statistical Power for GLN Reconstruction

Given the number of time points on a trajectory and the sample size per time point, one is statistically limited in detecting true interactions in a GLN beyond a certain network complexity by the statistical power. The gtts, distributions of each variable, sample size (number of replicas and time points), Type I error, and effect size together determine the statistical power. Power is independent of the computational approach used to reconstruct a GLN from observed trajectories. With estimation of statistical power, one can answer the question of whether the amount of data in the trajectory can statistically support any GLN for certain complexity at all.

Without loss of generality, we assume that the outcome of each entry in a gtt is a binomial variable. The same procedure below can be applied to a multinomial distribution. The success rate of a binomial variable is directly related to the strength of an interaction between the corresponding entry index (a specific parent combination) in the gtt and the binomial variable. When the success rate is 0.5, the specific entry has no better indication of the outcome of the binomial variable than mere chance; when the success rate is 0 or 1, this entry can always predict the outcome of the binomial variable correctly with probability 1. Thus, success rate 0.5 suggests no interaction between the entry index in the gtt and the binomial variable; success rate 0 or 1 suggests the strongest unambiguous interaction possible. We consider a true interaction existent when the success rate is not 0.5. Thus, a hypothesis testing against success rate 0.5 can be used to test against no interaction between an entry index in the gtt and the binomial variable. To study the power of such a test for an interaction (success rate ), we design the alternative hypothesis to be a binomial distribution with success rate , versus success rate  under the null hypothesis. The choice of 0.8 instead of 1 allows the relation to carry uncertainty, typically due to unexplained biological variation and technical noise inherent to experimental procedures used to develop biological data sets. The effect size is . In order to calculate the power, an effect size must be specified [24], as different values of  have different power. The test is two sided because  with an effect size of −0.3 is considered the same strength of interaction as . When the effect size changes, the qualitative change in power can be predicted. For example, if , the power will be lower than that of ; if , the power will be higher than that of . The Type I error rate  is adjusted to  considering multiple testing effect. Let  and  be the decision boundary. If  or , reject the null hypothesis, or equivalently the rejection region is  and , where  is the total number of trials. The decision boundaries  and  are determined such that (5)

where the binomial distribution is defined as (6)

The statistical power is (7)

**Figure 3:**
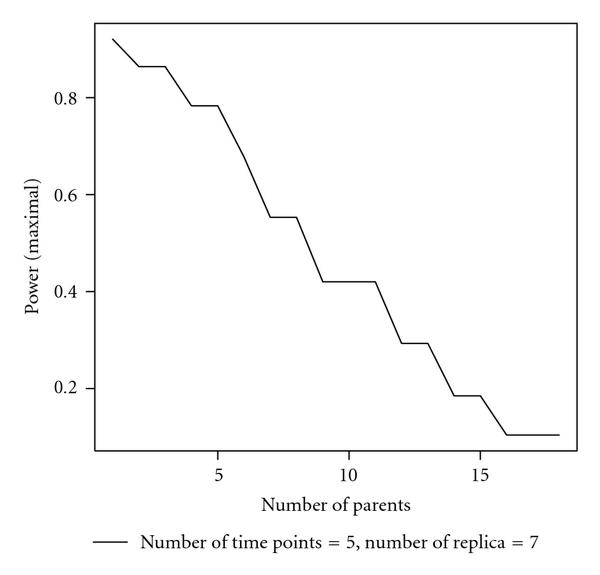
Statistical power for detecting a generalized logical network as a function of its network complexity, given number of time points (5), number of replicas per time point (7), network size (20), and hypotheses  (alternative) versus  (null).

Figure 3 plots the maximal power as a function of the network complexity of a GLN given the length of a trajectory and the number of replicas at each time point. The curve demonstrates that the more complex the network is, the lower the statistical power is, under the same experimental conditions. A (maximal) 68% power is possible if we use 5 time points for each condition with 7 replicas at each time point with a network of 20 genes, a complexity of 6, at a Type I error rate of 0.05. For a typical statistical power cutoff of 60%, our microarray experiment in Section 6 was justified. The Type I error  adjustment may be conservative as dependency may exist among time points. Although the binomial distribution can be replaced with a multinomial one in the gtt to calculate the statistical power, this study establishes the minimal requirements.

## 4. GLN Reconstruction through Multinomial Tests

A GLN can be reconstructed from observed trajectories of a system under perturbed conditions. There are two important issues in GLN reconstruction. The first one is how to search efficiently for the best among feasible GLN candidates. This issue depends on how one handles the combinatorial computational cost, generally *NP*-hard, incurred by reconstructing a GLN. The second issue is how to determine the false-positive rate that the best candidate arises out of randomness caused by noise and sampling errors in a network where no nodes interact, recently gaining attention such as in BN fitting [25]. Various criteria for goodness of fit have been used in reconstruction of a GLN from observed trajectories. Mutual information among variables has been employed in interaction graphs [26]; likelihood and BIC are used to determine network structure for Bayesian networks [27] and DBNs; the coefficient of determination has been used for BNs [20]. These measures, however, do not control the false-positive rate directly.

By performing multinomial tests on the transition tables at each node, we are able to resolve simultaneously both issues above in one framework. The network topology inference reduces to selecting the parents for each node through multiple applications of the same multinomial test. The false-positive control is achieved by setting an -level, which can be adjusted for multiple comparisons, for the tests at each node, instead of always keeping a parent selection with the best value of criterion as in all other approaches mentioned above. Our criterion is the statistical significance of each test. Thus, we move forward from existing network topology inference approaches by assessing the probability of false-positive interactions arising by chance in GLN reconstruction.

Table 2 shows the transition table of a single node , which can also be considered a contingency table. The number of rows in the table is .  is the number of observations in which the parents take the values in the th row at , and  takes the value of  at . Let  be the sum of column . Let  be the sum of row . Let  be the total number of observations. The following hypothesis test is designed for each row.

**Table 2:** The transition table of node

row						
				#0		
0	0		0			
1	0		1			
						
	1		2			

Null Hypothesis.

Alternative Hypothesis.

This hypothesis test determines if  is associated with parent values in row , in essence a multinomial test with the probability parameters,(8)

A multinomial test for row  inspects the chi-square statistic (9)

where (10)

is the expected count. Asymptotically,  has a chi-square distribution with  degrees of freedom.  can be computed for each row  in the table. By properties of the chi-square distribution, a summation of independent chi-squares is still a chi-square whose degrees of freedom are the summation of each individual's degrees of freedom. However, when we sum up all  over , we loose  degrees of freedom because each column has a fixed total. Thus, the transition table statistic(11)

is a chi-square distributed with (12)

degrees of freedom. We attach subscript  to  and  and let  with degrees of freedom  be the statistic for the transition table of the th node. We define the test statistic for a GLN with  nodes as (13)

Under the null hypothesis of no interaction,  are all independent. Thus,  has a chi-square distribution with  degrees of freedom by summing up  degrees of freedom for each transition table, that is, (14)

A -value can be computed for  to indicate the statistical significance of a GLN model. The -value provides a means to tradeoff between goodness of fit and complexity. Therefore, GLN reconstruction is to find a GLN with the minimum -value. Since the  statistics for the transition tables at each node are independent of each other, minimization of the overall -value reduces to minimizing the -values for individual transition tables at each node.

Once an optimal set of transition tables at each node are identified, gtts can be derived by maximum likelihood estimation of probabilities for the multinomial distribution on each row. Each row is assigned a truth value that corresponds to the maximum probability parameter in its multinomial distribution. Although not implemented in this paper, a probabilistic GLN can be reconstructed, not by setting a gtt, but by keeping the probability parameters in the multinomial distribution for each row. The GLN reconstruction algorithm is presented as Algorithm 1 Reconstruct-GLN. It searches an optimal gtt that minimizes the -value with up to  parents for each node. The time complexity of the algorithm is (15)

where  is the maximum quantization level of all nodes.

**Algorithm 1:**Reconstruct-GLN (A collection of observed trajectories, -level, ).

**For** each node **do**

* ***For** to **do**

*  ***For** each possible selection of  parents **do**

*   *Accumulate a transition table from given trajectories

*   *Compute -value by performing multinomial test on the transition table

*   ***if**-value is smaller than the current minimum -value for the current node **then**

*    *minimum -value -value

*    *Record the current transition table

*    *Replace previous parents with the current selection of  parents

*   ***end if**

*  ***end for**

* ***end for**

* *Perform -value adjustment for multiple comparisons involved in parent selection

* ***if** the adjusted -value is less than the given -level **then**

*  *Convert the transition table with the minimum -value to a gtt by maximum likelihood

*  *estimation of multinomial parameters

* ***else**

*  *Declare that the current node has no parents

* ***end if**

end for

 Compute the overall -value for the reconstructed GLN

 Return the reconstructed GLN, the associated -values for each node, and the overall -value

## 5. Accuracy of GLN versus DBN Reconstruction

As GLN modeling is proposed as a potential alternative to DBN modeling, it is important to assess the performance of GLN relative to DBN modeling in terms of their abilities to recover the topology of the underlying networks. We use Hamming distance, false positives, and false negatives to evaluate the difference between a reconstructed network and the original ground-truth network. The Hamming distance is defined by the total number of different directed edges between two networks of the same set of nodes. A false positive is an incidence of a directed edge in the reconstructed network but not in the original ground-truth network; a false negative is an incidence of a directed edge in the original network but not in the reconstructed network. The definitions imply that the Hamming distance is the sum of false positives and false negatives. We have chosen to use a simulated data set over a real biological data set, such as the yeast cell cycle gene expression data set, to do the performance evaluation. This is because many factors in a biological data set may contribute to the reconstruction performance in addition to the algorithm difference. For example, the ground truth GRN in yeast may not contain all active interactions; it may also include additional interactions that are inactive in the particular experiments. This makes the comparison of algorithm performance less certain. In a simulated example, one has control of all potential variations.

Under the Markovian and some other noise assumptions, DBN reconstruction can be reduced to the maximum likelihood estimation of the conditional distributions of each node. In the discrete variable case, the conditional distributions are multinomial. In DBN reconstruction, the BIC defined by (16)

is often evaluated to balance maximum likelihood estimation with the number of parameters in each conditional distribution. In contrast, the  statistic is used in GLN modeling, as opposed to the likelihood in DBN modeling; the tradeoff with model complexity in GLN modeling is incorporated into the degrees of freedom of the  distribution, as opposed to the  term in the BIC in DBN modeling. Additionally, GLN modeling allows the user to control false-positive rate by specifying the size  for type I error, while DBN modeling does not facilitate such an option.

We first randomly generated 20 first-order Boolean networks, each consisting of 10 nodes with a maximum of two parents per node. We simulated the dynamics of each Boolean network by calculating trajectories starting from a random initial state with 25 steps (26 time points in total). Then, we randomly flip each value with probability  in the trajectory with the following noise model: (17)

For each trajectory, we applied increasing levels of noise with . When , the noise is the strongest in terms of network topology reconstruction. When , it is the same as  as far as the topology is concerned.

**Figure 4:**
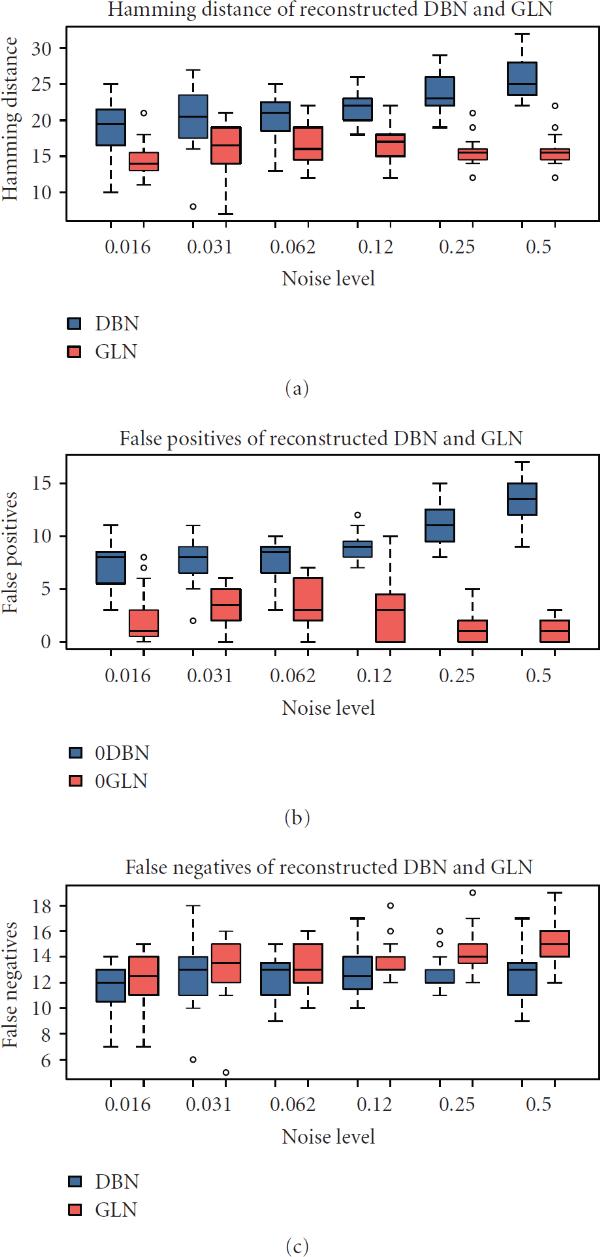
**Performance comparison between generalized logical network and dynamic Bayesian network modeling, including the boxplots of Hamming distance, false positives, and false negatives as functions of increasing noise level (flip probability )**.

The performances of GLN ( level at 0.05 with -values adjusted) and DBN are shown in Figure 4. The Hamming distance, false positives, and false negatives are plotted as functions of increasing noise levels (flip probability ). The lower the Hamming distance, the similar the reconstructed network to the original one. GLN modeling definitely has consistently smaller Hamming distances and less variance under various levels of noise than DBN modeling. This Hamming distance advantage of GLN over DBN attributes mainly to the fewer false positives of the GLN reconstructions. Although the average false negatives of GLN are slightly higher than DBN, the difference is not strongly statistically significant. Overall, the GLN reconstruction performs consistently better than the DBN reconstruction. This example to some extent establishes that GLN modeling is promising for further study and development.

GLN modeling is built on statistical hypothesis testing, while DBN modeling on information theory. We are curious at a more theoretical level why the GLN reconstruction has shown a consistently superior performance over the DBN reconstruction in the simulation study. We plan to address this remaining issue in our future work.

## 6. Temporal Gene Expression in Mice Exposed to Alcohol

Thirty-five adult DBA/2J (D2) mice were housed on a 12:12 light:dark cycle and given food and water ad libitum. The mice were habituated for three days to i.p. injections of saline and on the forth day were injected with 20% alcohol in saline in a total dose of 4 g/kg. D2 mice are exquisitely sensitive to alcohol dependence, and at this dose show physical signs consistent with dependence from about 4–10 hours after injection. Brains were removed, and anterior cortex tissue was dissected at 2, 7, 12, and 24 hours following the alcohol injection with 7 biological replicates at each time point. All animals were housed and treated according to the National Institutes of Health guidelines for the use and care of laboratory animals [28] and an approved Institutional Animal Care and Use Committee protocol.

cDNA fragments, that had undergone PCR from clones, were printed on poly-L-lysine-coated (Sigma, Mo, USA) microscope slides (Erie Scientific, Portsmouth, NH, USA) using a custom-built robotic arrayer as described in [29]. The clones were from several cDNA libraries, including ESTs cloned in the laboratory of S.E.B., Research Genetics/Invitrogen clone sets Brain Molecular Anatomy Project and Sequence Verified, and the National Institute on Aging (3) clone sets 7.4 K and 15 K. cDNA microarrays were hybridized using the 3DNA array 900 microarray labeling kit according to the manufacturer's protocol (Genisphere, Hatfield, Pa, USA). Total RNA samples were reverse transcribed, labeled with Cyanine-3 (Cy-3), and hybridized against a common reference RNA labeled with Cy-5. The common reference is whole-brain RNA extracted from 100 male B6 mice. All arrays contained the same reference RNA in the Cy-5 channel and were normalized by using within-print tips Lowess nonlinear normalization [30]. Normalized array data were stored in the longhorn array database (LAD) [31] and then standardized by using the red channel (common reference RNA) as the baseline standard with software developed in the laboratory of S.E.B. (These PERL programs are available upon request.) Data were loaded into an in-house database used for sorting by various statistics.

## 7. GLN Modeling of Transcription Regulation in the Mouse Brain

We demonstrate a GRN reconstructed using GLN modeling from a microarray study of temporal gene expression microarrays in mouse brains following acute exposure to alcohol to uncover transcription interactions of involved genes. The microarray data were normalized, quantized, formed to trajectories, and used to reconstruct a GLN. We illustrate the significant interactions we identified, their agreement with the literature, as well as the dynamic behavior of the GRN in response to alcohol.

Through post hoc -tests, partial least squares, and one-way ANOVA (fixed effect only and  without multiple testing correction) across time course analyses, a total of 392 differentially expressed genes were selected because they exhibit both temporal and alcohol related expression variation. Missing gene expression values were imputed using the R software package PAMR [32]. Those genes not selected for inclusion do not have strong evidence from this experiment to be on any path from the alcohol node.

**Figure 5:**
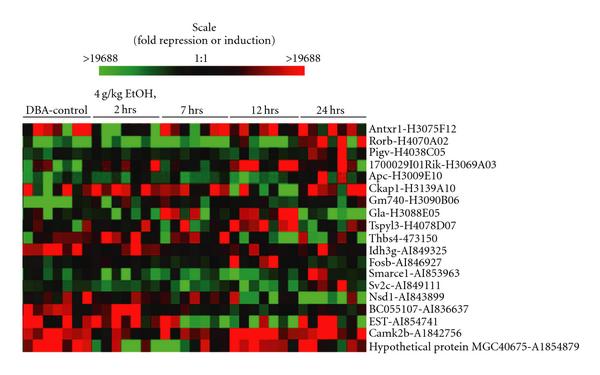
**Expression of the 19 selected genes**. Microarray results are shown in pseudo color raster display. Each column represents an array of a single mouse and the rows show expression for a given gene. Transcripts for which expression is increased are plotted in green and for which expression is decreased are plotted in red. From left to right are control and 2, 7, 12, and 24 hour time points following a single 4 g/kg i.p. injection of alcohol (7 replicates per time point).

Among the 392 selected genes, we performed maximum likelihood joint quantization [33, 34] to obtain a list of 19 genes for GLN modeling. The multidimensional quantization algorithm aims at finding a grid to preserve interactions during the discretization. A variable is quantized only to finer levels if doing so captures its interaction with other variables. The quantization levels for each dimension were automatically chosen between 1 and 4. Thus variables receiving no more than one quantization level lack interactions with any other variables and are filtered out. There are three major steps in the quantization. The first step is to initialize with a finest possible grid—a line is added between every pair of consecutive points in each dimension. The second step is to remove a grid line one by one as long as the performance (joint likelihood penalized by the total number of grid lines) improves. The third step is to finalize the grid when the performance starts to suffer as a result of removing grid lines further. It is critical for the quantization to preserve the interactions among the original continuous random variables; otherwise the ensuing GLN modeling would not be informative if interactions are destroyed or invented by a less intelligent quantization method. After quantization was applied, 19 genes ended up with exactly 2 quantization levels, while the remaining 373 genes were all quantized to a single level and thus filtered out for further modeling. The expression patterns of these 19 genes are shown in Figure 5.

These selected genes were entered into the GLN model as candidate GLN components that connect to the alcohol treatment node through gene expression on a directed path.

The alcohol node is assigned based on the experimental condition: 1 for alcohol-injected samples and 0 for control samples. The quantization was implemented in Java and compiled to native code on SuSE Linux using the GCJ compiler. It took about 5 hours to finish the quantization on a 2.8 GHz Pentium dual-core processor computer with 4 GB RAM running SuSE Linux.

**Figure 6:**
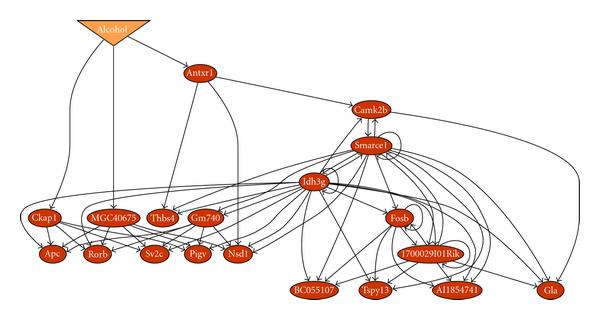
**An inferred generalized logical network (-value )**. The oval nodes represent genes and the inverse triangle, the binary value of alcohol treatment or control for each subject.

**Figure 7:**
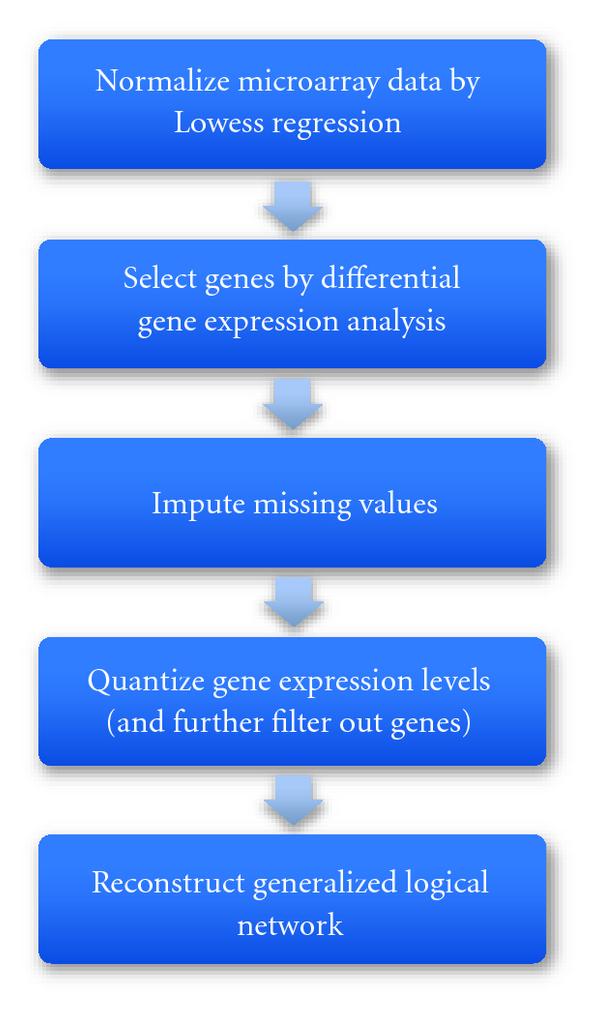
**Five major steps in the entire modeling process from raw gene expression time course data to a generalized logical network model of a gene regulatory network**.

From the preprocessed and quantized temporal gene expression data, we reconstructed a GLN as shown in Figure 6. The size of the statistical test in the reconstruction was 0.05. The maximum number of parents per node is 6. The overall -value of the reconstructed GLN is , and the -values for gtts at each node are given in Table 3. The GLN reconstruction software was written in C/C++. It was tested on trajectories from known GLNs, recovered the trajectories correctly, and returned GLNs identical to or simpler than the true ones. The program took about 4.5 hours to complete GLN modeling of the 20 node data (19 genes plus an alcohol node) on a 2.8 GHz Pentium dual-core processor computer with 4 GB RAM running SuSE Linux. The entire modeling process is summarized by the flow chart in Figure 7.

**Table 3:** The -values and number of parents for each node in the generalized logical network

Node	Symbol	No. of parents	-value
1	Alcohol	—	—
2	Idh3g	2	0
3	Rorb	4	2.9e-15
4	AI854741	4	0
5	Nsd1	5	0
6	Gla	4	0
7	Camk2b	3	4.4e-12
8	Sv2c	4	0
9	Fosb	4	0
10	Gm740	2	3.1e-14
11	MGC40675	1	5.0e-15
12	BC055107	4	2.1e-10
13	Tspyl3	4	0
14	1700029I01Rik	4	0
15	Smarce1	4	3.5e-15
16	Antxr1	1	3.9e-11
17	Pigv	4	0
18	Thbs4	3	0
19	Ckap1	1	5.7e-07
20	Apc	4	1.4e-13

**Figure 8:**
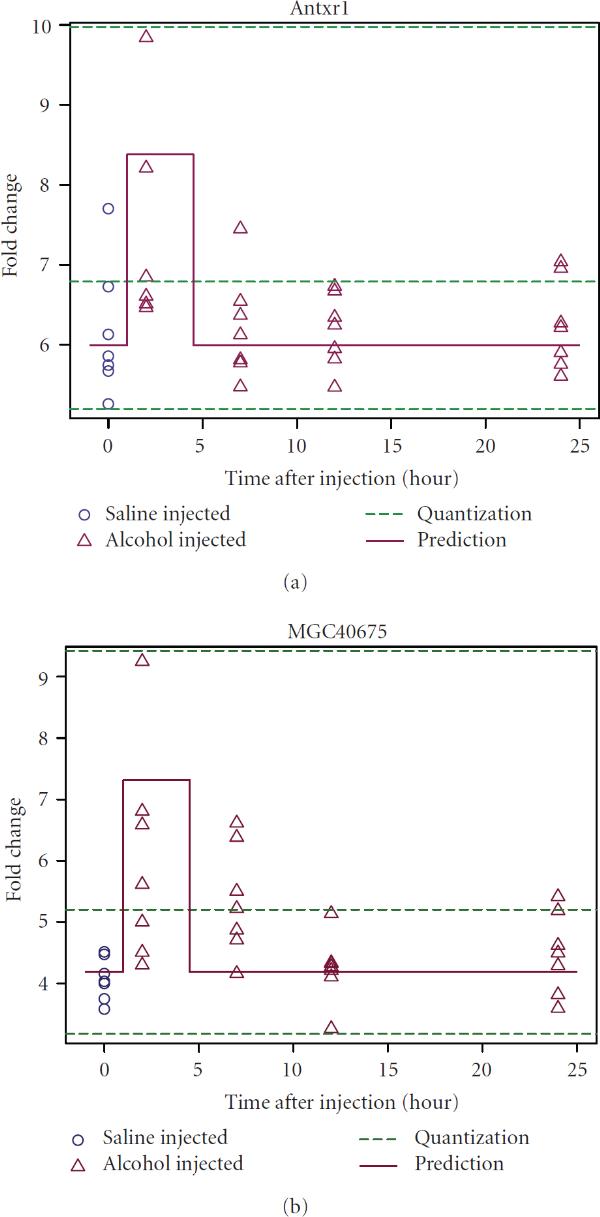
**Agreement of model predicted time courses with observations**. Trajectories (solid lines) are predicted from the reconstructed generalized logical network model under the alcohol condition, shown with the observed time course (circle: saline injected, or control; triangle: alcohol injected). The quantization to convert the original continuous fold changes to discrete ones is also displayed as the dashed lines. Both genes showed consistent dynamics between the model prediction and the observation in response to alcohol and are central nodes in the reconstructed gene regulatory network.

As a GLN model has precisely defined transition logics associated with each node, one can predict the dynamics of the underlying system and assess the accuracy of the model. Figure 8 demonstrates how the reconstructed GLN model of the interactions may have captured the consistent behaviors shown in the time courses in response to alcohol. Both genes shown (*Antxr1* and *MGC40675*) respond to the injection of alcohol sharply after 2 hours of injection. However, they both return to normal levels after 24 hours of exposure. Although the predicted trajectories cannot capture all subtle changes in the original time courses, the prediction agrees with the overall trend in the observation. This suggests that the model fitting preserved the dynamics in both genes.

**Figure 9:**
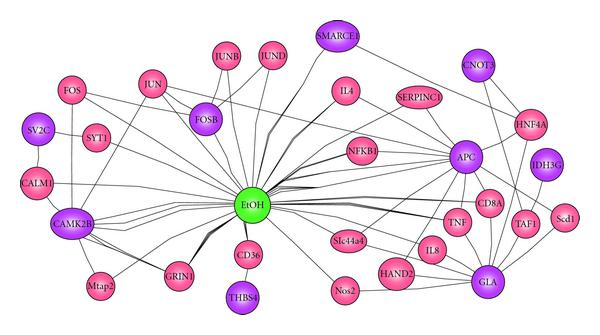
**Genes responsive to alcohol (the EtOH node) uncovered by PathwayArchitect from literature**. The purple nodes were identified in Figure 6.

In this GLN (Figure 6), *Idh3g, Smarce1, 1700029I01Rik, Gm740, MGC40675, Fosb, Ckap1*, and *Camk2b* are the most influential gene nodes. It should be noted that not all of the genes that were identified as network members are part of the conventional transcriptional regulatory system. The genomic approach employed in these studies enables detection of broader modifiers of transcription, including those genes which are involved in neuronal processes which in turn result in altered transcriptional activity. In fact, major neural pathways are represented. The interactions with alcohol for *Smarce1* [35], *Fosb* [36], and *Camk2b* [37] are biologically verified. In addition, nine out of the 19 nodes in our GLN (Figure 9) have been identified as interacting with alcohol from biology literature by PathwayArchitect (Stratagene, La Jolla, Calif, USA). From another literature database tool Ingenuity Pathway Analysis (INGENUITY SYSTEMS, Redwood City, Calif, USA), we have found nine genes, *Antxr1, Thbs4, Rorb, Smarce1, Nsd1, Bc055107, Camk2B, Gla*, and *Fosb*, on the major canonical hepatic cholestasis, PPAR signaling, and xenobiotic metabolism signaling (e.g., *Camk2b*) pathways. The PPAR pathway is involved in the alcoholic metabolism. This indicates that our approach was indeed successful in capturing significant causal interactions through temporal dependencies. More importantly, however, new hypotheses for several genes that had never before been implicated in alcoholism were generated. Without a model which has the ability to detect statistically significant interactions, these would not otherwise have gained attention. Some of these putative network members and relations may be false positives. The molecular mechanisms of alcoholism are complex. Alcohol is a dirty drug, meaning that it acts on a diverse range of neurological processes. Its mechanisms of action are still poorly understood at the gene expression level, as this is a relatively new and active area of investigation in the alcohol research field. Most of the genes we report have not been associated with alcohol responses to date. The ability to contribute novel data-driven hypotheses to this research area will facilitate the planning of future studies, for example, in prioritizing which of over 45,000 proposed new knock-out mice [38] to rederive and test for phenotypic effects related to alcohol response. Ultimately, confirmatory validation experiments and convergent evidence from other high throughput molecular analyses are essential. These results demonstrated that our algorithm can generate and prioritize new hypotheses for understanding complex traits such as alcoholism.

**Figure 10:**
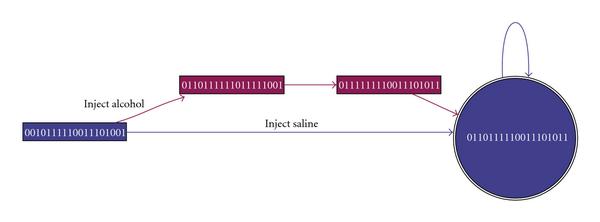
**The state transition diagram and attractor cycles of the inferred gene regulatory network**. A square node stands for a transient state; a round one, an attractor. Inside each node, a sequence of numbers indicates the state of all genes that node represents. A directed edge from a source to a destination node suggests that the state represented by the source node evolves into the state by the destination node. The red color encodes the states under the influence of alcohol; the blue color saline (control).

Through simulation of the reconstructed GLN, a state transition diagram corresponding to the GLN is shown in Figure 10. Beyond the detected associations with alcohol in the GLN, a possible dynamic mechanism is portrayed in this diagram. The figure reveals that expressed genes eventually merge into the same attractor cycle or steady state after injection of alcohol (marked by red) and saline (the control, marked by blue). This can be interpreted to reflect a restoration of normal expression levels following acute exposure. This additional information cannot be readily discerned from the GRN in Figure 6, but is apparent from the transition diagram in Figure 10. It thus suggests that injection of alcohol in the D2 mouse strain does not result in lasting change in the expression profile for these genes and rather has produced a transient effect on the behavior of the GRN. Biologically, one would expect most of the changes to return to "normal" as the last time point is at 24 hours and all alcohol is gone—the withdrawal symptoms have returned to the baseline. In another study of a chronic alcohol exposure with a longer, three day, "drunk time" after multiple alcohol injections, we observed similar expression patterns in the mouse brain tissue.

## 8. Conclusions and Future Work

Derived from a statistical property regarding the summation of independent chi-squares, our GLN reconstruction algorithm identifies significant dynamic associations among a subset of genes to a target gene by performing the multinomial test. Thus, we have offered a unique framework to reconstruct GLNs to characterize temporal interactions from time-course gene expression data. Results from our application of this technique to the study of alcohol's influence on gene expression in mouse brains reveal both consistently observed associations and novel hypotheses that remain an open problem for current biological investigation. Based on these results, there appears to be significant potential to inspect the temporal patterns in gene expression through GLN reconstruction. In this paper, we have demonstrated the value of GLN modeling for extracting the underlying causal interactions among genes involved in response to alcohol. Some of the inferences made on temporal dependencies corroborate present knowledge on gene regulation in mouse. The other inferences will be subject to more extensive in vivo biological verification.

Preselection of a subset of interesting genes to render a model computable is a challenge for GRN modeling from microarray data. Approaches which filter genes or gene-gene relations have been applied. While this leads to the improved signal in the data, it also introduces a problem of false-negative results, neglecting extensive information on highly relevant genes which exhibit subtle variation in the same temporal patterns as other connected genes. Rather than filtering based on statistical effects, one could develop GLN models from known pathways and evaluate how they respond and interact with pharmacological perturbations. This strategy can be implemented by reconstructing GLNs from GRNs established by literature mining such as Ingenuity Pathways Knowledge Base (size Ingenuity Systems, Redwood City, Calif, USA) and PathAssist (size JusticeTrax Inc., Mesa, Ariz, USA). This will possibly allow the modeling to begin at a more realistic starting point, and will reserve statistical power for the strong plausible relations that are previously reported.

A more diverse set of nodes can also be incorporated into the GLN modeling. The biological relevance of a reconstructed GLN can be substantially improved if simultaneous measurements of the proteome, the metabolome, and the transcriptome are available, without major modifications to the current algorithms. Once data are properly scaled, the method is highly generalizable and has significant potential for inferring temporal relations among widely diverse biological processes. The illustration of the validity of our results from a small time-course gene expression study indicates substantial potential for denser sampling, and for the incorporation of additional data representing other aspects of the neurobiological response to alcohol, including neurohormonal, physiological, and behavioral measures.
